# Moving away from the "unit cost". Predicting country-specific average cost curves of VMMC services accounting for variations in service delivery platforms in sub-Saharan Africa

**DOI:** 10.1371/journal.pone.0249076

**Published:** 2021-04-22

**Authors:** Sergio Bautista-Arredondo, Carlos Pineda-Antunez, Diego Cerecero-Garcia, Drew B. Cameron, Lily Alexander, Chris Chiwevu, Steven Forsythe, Michel Tchuenche, William H. Dow, James Kahn, Gabriela B. Gomez, Anna Vassall, Lori A. Bollinger, Carol Levin

**Affiliations:** 1 Center for Health Systems Research, National Institute of Public Health, Cuernavaca, Mexico; 2 University of California Berkeley, Berkeley, CA, United States of America; 3 Department of Global Health, University of Washington, Seattle, WA, United States of America; 4 Independent Consultant, Fairfax, VA, United States of America; 5 Avenir Health, Glastonbury, CT, United States of America; 6 Philip R. Lee Institute for Health Policy Studies, University of California, San Francisco, CA, United States of America; 7 Department of Global Health and Development, London School of Hygiene and Tropical Medicine, London, United Kingdom; Columbia University - MSPH, UNITED STATES

## Abstract

**Background:**

One critical element to optimize funding decisions involves the cost and efficiency implications of implementing alternative program components and configurations. Program planners, policy makers and funders alike are in need of relevant, strategic data and analyses to help them plan and implement effective and efficient programs. Contrary to widely accepted conceptions in both policy and academic arenas, average costs per service (so-called "unit costs") vary considerably across implementation settings and facilities. The objective of this work is twofold: 1) to estimate the variation of VMMC unit costs across service delivery platforms (SDP) in Sub-Saharan countries, and 2) to develop and validate a strategy to extrapolate unit costs to settings for which no data exists.

**Methods:**

We identified high-quality VMMC cost studies through a literature review. Authors were contacted to request the facility-level datasets (primary data) underlying their results. We standardized the disparate datasets into an aggregated database which included 228 facilities in eight countries. We estimated multivariate models to assess the correlation between VMMC unit costs and scale, while simultaneously accounting for the influence of the SDP (which we defined as all possible combinations of type of facility, ownership, urbanicity, and country), on the unit cost variation. We defined SDP as any combination of such four characteristics. Finally, we extrapolated VMMC unit costs for all SDPs in 13 countries, including those not contained in our dataset.

**Results:**

The average unit cost was 73 USD (IQR: 28.3, 100.7). South Africa showed the highest within-country cost variation, as well as the highest mean unit cost (135 USD). Uganda and Namibia had minimal within-country cost variation, and Uganda had the lowest mean VMMC unit cost (22 USD). Our results showed evidence consistent with economies of scale. Private ownership and Hospitals were significant determinants of higher unit costs. By identifying key cost drivers, including country- and facility-level characteristics, as well as the effects of scale we developed econometric models to estimate unit cost curves for VMMC services in a variety of clinical and geographical settings.

**Conclusion:**

While our study did not produce new empirical data, our results did increase by a tenfold the availability of unit costs estimates for 128 SDPs in 14 priority countries for VMMC. It is to our knowledge, the most comprehensive analysis of VMMC unit costs to date. Furthermore, we provide a proof of concept of the ability to generate predictive cost estimates for settings where empirical data does not exist.

## Introduction

HIV continues to be a major public health challenge with global repercussions for domestic health systems and international donor agencies [1]. In 2016, an estimated 36.7 million people were living with HIV, [2]. In the same year, there were 1.7 million new HIV infections, with more than two-thirds occurring in sub-Saharan Africa [1].

Strategies to prevent HIV transmission are imperative to continue progress toward reducing HIV infections. Program planners, policy makers and funders alike are in need of relevant, strategic data and analyses to help them plan and implement effective and efficient programs. Maximizing health benefits given the increasing insufficiency of available funds is now more urgent than ever.

One critical element to optimize funding decisions involves the cost and efficiency implications of implementing alternative program components and configurations. Contrary to widely accepted conceptions in both policy and academic arenas, average costs per service (so-called "unit costs") vary considerably across implementation settings and facilities [3–7]. The factors behind this variation are complex and include both justifiable and systematic causes, as well as health system inefficiencies and waste. Yet, most of the policy-oriented literature on costs and cost-effectiveness continues to rely on the conception of one single unit cost to make recommendations, plan scaling-up of programs, and assess efficiency. As a consequence, decision-makers continue to make policy decisions based on often misconceived and flawed cost data and assumptions.

The policy relevance of cost information hinges on its accessibility and availability in a timely fashion. However, costing studies that appropriately measure costs and assess cost variation are scarce. Additionally, funding and time limitations restrict the type, quality, and quantity of studies. And yet, the need for such information is growing as global funding for HIV programs shrinks and rates of new HIV infections remain stubbornly high [8].

A strategy is needed to address these challenges. The objective of this work, which is part of the Global Health Cost Consortium (GHCC) initiative [9,10], was to use facility-level cost data to model unit costs curves of Voluntary Medical Male Circumcision (VMMC) services for a variety of implementation settings. Specifically, our aim is twofold: 1) to explicitly account for the effect of service delivery platforms on the variation of VMMC unit costs and 2) to develop a strategy to extrapolate unit costs to settings for which no data exists.

## Methods

### The Global Health Cost Consortium

The Global Health Cost Consortium was a three-year project funded by the Bill and Melinda Gates Foundation which aimed to increase the efficiency and effectiveness of HIV and TB prevention and treatment by curating and facilitating access to available cost data, and producing standards for future cost data collection and analysis. As part of the GHCC objectives, we intended to inform the development of methods to estimate unit costs of various interventions and settings, while explicitly accounting for critical parameters describing service delivery platforms and delivery settings. Ultimately, our approach seeks to produce vital information to program planners, policymakers, funders and academics.

### Voluntary medical male circumcision

Voluntary medical male circumcision has been shown to be an effective intervention to reduce HIV transmission [11,12]. Several studies, including randomized controlled trials (RCT), have demonstrated that VMMC reduces HIV transmission by up to 60% and decreases the risk of other sexually transmitted infections (STI) in men and their female partners [11,13–15]. When combined with other evidence-based prevention and treatment methods, studies have shown that VMMC reduces new HIV infections even further [16].

Based on the well-documented benefits of VMMC, the World Health Organization (WHO) has recommended the scale-up of VMMC in high HIV prevalence countries. In 2011, the WHO and UNAIDS set a target of reaching 80% VMMC coverage for adult males in 13 priority African countries by 2015, including Botswana, Kenya, Lesotho, Malawi, Mozambique, Namibia, Rwanda, South Africa, Swaziland, Uganda, Tanzania, Zambia, and Zimbabwe [17]. While some of these countries are close to the target, not all of them have yet reached the threshhold [18]. Furthermore, recent investment cases for preventing HIV and policy analyses to support Universal Health Coverage interventions, include VMMC as part of an essential package of interventions [19,20].

### Overview of methods

We developed a multi-stage approach using several data sources. First, we identified relevant VMMC costing studies through a systematic review of the literature [9]. Second, we contacted authors of selected papers to request the facility-level datasets (primary data) underlying their results, while simultaneously extracting cost estimates (secondary data) from all publications. Third, we standardized both primary and secondary data for joint analyses. Fourth, we modeled VMMC costs curves to predict the relationship between unit costs and scale for distinct service delivery platforms. Finally, we extrapolated VMMC cost curves for service delivery platforms outside our sample of countries.

### Identifying VMMC studies

Our anslysis uses empirical data from twenty-nine VMMC costing studies identified through an extensive literature review [9]. The initial systematic search included several HIV interventions (e.g., VMMC, antiretroviral treatment, pre-exposure prophylaxis, etc.). For this analysis, we selected only VMMC costing studies. The systematic search of databases was conducted for studies published between January 2006 and October 2017, and included Web of Science, Cochrane, PubMed, Embase, NHS EED, LILACS, Google Scholar, several gray literature resources and snowball searching. The initial search yielded 23,938 documents. Of these, 2,566 published and gray literature studies were identified and assessed for inclusion in the study. We identified 58 studies reporting costing information on VMMC interventions. Finally, we selected 29 studies for inclusion after removing papers reporting modeled cost data, those with insufficient or incomplete costs results, or those with duplicate information.

### Requesting data

We compiled data sets from multiple studies directly from their authors. Following a standard protocol (S1 Fig), we requested primary data from reports and papers which met the following additional inclusion criteria: i) facility-level data and ii) sample size greater than one site. Of the twenty-nine original VMMC studies, fifteen met these additional inclusion criteria. We contacted two authors from each paper to request the underlying primary data; typically, the corresponding and the first authors. The authors from eight studies declined to collaborate or did not answer, narrowing our final primary data sample to seven studies, comprising 238 facilities across eight countries.

Of the 238 facilities, 220 had complete information. The studies covered the years 2008 to 2013, with various data collection dates. We requested the following data elements for each paper, at the facility level: total cost and total cost disaggregated by input categories (personnel, capital, recurrent and other); total unit cost and unit cost disaggregated by cost categories; outputs (total annual number of male circumcisions performed); facility characteristics including facility type, ownership, and urbanicity; and outreach models, i.e., fixed (facility-based) vs. outreach (mobile services).

### Extracting data from published costing studies

Extraction of data from published and gray literature articles was conducted using a standardardized strategy across all interventions and disease areas and is explained in greater detail elsewhere [9]. Breifly, all VMMC studies identified during our sysetmatic search and screening process were dividied among three extractors who recorded relevant cost and study attribute information and recorded it into a common format with each observation (row) representing a unique VMMC unit cost. Version control was managed by a lead data manager, each variable was examined for outliers that might suggest potential transcription errors and each observation was double-checked for accuracy and quality by a senior researcher. Any discrepancies were reconciled by the original extractor, lead data manager and senior researcher.

### Standardizing data

#### Primary data

Primary data consisted of disparate datasets received directly from study authors which we combined into an harmonized dataset. We used a standardization process as follows: 1) We transfered each dataset into a flat (horizontally oriented), facility-level dataset; 2) we filled-in a study-specific codebook with critical variables; 3) we standarded the input cost categories across data sources; 4) we concatenated the separate cleaned datasets into one final analytic dataset. All analyses were conducted using Stata SE version 15. See more details on S1 File, Standardization Process.

All the cost data were converted from local currencies to United States dollars (USD) using exchange rates reported by the World Bank according to the year of data collection. We inflated all costs to 2016 using the gross domestic product (GDP) Implicit Price Deflator methodology [21].

#### Secondary data

We extracted published unit cost data from the literature review to create a complementary data set, hereafter referred to as ’secondary data.’ We standardized secondary data previously extracted from publications for those studies whose authors either declined to share their primary data, did not respond to our emails, or did not meet inclusion criteria. As with primary data, secondary data were standardized by exchanging local currencies to US Dollars and then inflated to 2016 USD using the United States’ GDP Implicit Price Deflator [21].

Authors typically reported a single unit cost reflecting the average unit costs across several sites (mostly facilities). In the case of the two studies from Kenya, the GHCC team extracted six unit cost observations which resulted from averages across 251 platforms throughout the country [22]. We excluded observations due to two main reasons: underestimation of the total unit costs due to missing input costs; i.e., studies that failed to measure critical inputs such as staff; and unit costs that resulted from averaging facility-level unit costs from a mixed sample of facilities regarding urbanicity, ownership or type of facility.

### Pooled data—Aggregation of data to the level of *service delivery platform*

For the final stage of the analysis we aggregated or *collapsed* the facility-level primary data to the level of SDP, in order to produce SDP-level exptrapolations. Our primary data set was reduced from 220 facility-level unit costs to 38 SDP-level unit costs after collapsing, plus an additional nine observations from two countries from secondary data. See S2 Fig. for a description of the process of collapsing data, and S1 Table for a list of SDPs. The pooled dataset was comprised of 47 unit costs derived from 16 different studies (seven primary studies and nine published papers).

### Defining service delivery platforms (SDP)

Overall, primary data included facility-level cost estimates for eight countries, across a wide range of delivery strategies and settings. We standardized the definitions of those setting characteristics according to three mutually exclusive categories:

- the type of facility (clinic or hospital),
- ownership (public or private), and
- urbanicity (urban or rural).

We then defined a *service delivery platform (SDP)* as any given combination of those three categories in each country in our sample. For example, one service delivery platform might be public, rural clinics in Kenya. Another would be private, urban hospitals in Nigeria; or public, rural hospitals in South Africa, and so forth. We were able to define 64 different service delivery platforms across eight countries. Secondary data comprised SDP-level estimates of unit costs across 6 countries, with a total of 9 observations (see S1 Fig).

### Definition of unit costs

To measure unit costs of VMMC, we included three broad input cost categories—capital, recurrent, and personnel. We defined capital costs as non-consumable goods lasting over one year, such as equipment and vehicles; recurrent costs as consumable supplies (e.g., HIV test and disposable circumcision kits), maintenance expenses, utilities, and training; and personnel costs as salaries of direct medical staff (physicians and nurses), and other non-medical staff (managers, supervisors, and ancillaries). The total cost of VMMC for each facility was defined as the sum of capital, recurrent and personnel costs. The VMMC unit cost, was the facility-level average cost per circumcision, i.e., the total annual cost VMMC divided by the total number of male circumcisions performed (See S1 File).

We also extracted the unit costs secondary data as reported in the publications. During the standardization process we confirmed that the descriptions of the unit costs definitions were consistent with ours. Given that the published estudies identified used a similar definition of unit costs, chages were not required.

### Analysis

#### Primary data analysis–unit cost curve

We used Ordinary Least Squares (OLS) and Generalized Linear Models (GLM) to model facility-level unit cost curves with respect to scale. In particular, we estimated multivariate models to assess the correlation between VMMC unit costs and scale, while simultaneously accounting for the influence of the type of facility, ownership, urbanicity, and country, on the unit cost variation in our sample.

The dependent variable in the OLS regression models was the natural logarithm of the facility-level average cost per male circumcision (unit cost). In GLM regressions we used the unit cost per male circumcision with a gamma family and log link function [23]. The independent variable was scale (number of VMMC per year), operationalized as a continuous independent variable. We also tested for non-linearities using higher order scale parameters.

To ensure that our regression coefficients were robust, we explored different model specifications. We controlled for urbanicity, ownership, type of facility, outreach, and 2016 GDP per capita. Based on the Akaike information criteria (AIC) and Bayesian information criteria (BIC) we selected the best-fitted model [24]. We also used the F-statistic to explore differences in model performance after removing non-significant variables. We also tested for heteroskedasticity in all models.

We explored the presence of multicollinearity with the variance inflation factor (VIF) to ensure OLS models were appropriate. We also performed sensitivity analyses using GLM models on the same regression specifications as OLS to test for robustness. The differences between OLS and GLM models were negligible. We present the final model specification we used in Eq 1:

$$ln{(UC}_{ic})=\alpha +\beta_{1}S_{ic}+\beta_{2}S_{ic}^{2}+\beta_{3}T_{ic}+\beta_{4}O_{ic}+\beta_{5}U_{ic}+\beta_{6}D_{ic}+\beta_{7}{GDP}_{c}+\beta_{8}Y_{ic}+\beta_{9}T_{ic}*O_{ic}$$ (1)

Where UC_*ic*_ is the average annual cost per VMMC performed in facility *i*, in country *c*. S_ic_ is the annual number of VMMCs performed in facility *i*, in country *c*. T_ic_ (facility type) takes the value 1 if hospital and 0 if clinic. O_ic_ (ownership) takes the value 1 if the facility is private and 0 if public. U_ic_ (urbanicity) takes the value 1 if the facility is located in a rural area and 0 if urban. D_ic_ (delivery model) equals 1 if outreach and 0 if the service was fixed. GDP_c_ is the 2016 GDP per capita in country *c*. Y_ic_ is the year of data collection for each study. In addition, we included an interaction term between facility type and ownership.

### Pooled data analysis—Extrapolation of unit cost curves

The goal of the second analytic approach was to predict VMMC unit costs for any SDP in 13 countries, including those not contained in our dataset. We followed a similar process to select the best model specification for extrapolating unit costs as the one we used for the unit cost curves analysis described above. We examined different specifications using both OLS and GLM, exploring non-linearities and interactions and compared their relative performance based on AIC and BIC criteria. We also tested for multicollinearity with the VIF parameter. In addition to including the variables defining the service delivery platforms–GDP per capita, type of facility, ownership, and urbanicity; we explored additional variables describing the context at the country level, including economic, health system, and epidemiological characteristics. The final specification used for the extrapolation analysis is defined by Eq 2:

$$ln({UC}_{sc})=\alpha +\beta_{1}{GDP}_{c}+\beta_{2}T_{sc}+\beta_{3}O_{sc}+\beta_{4}U_{sc}+\beta_{5}{Cov}_{c}+\beta_{6}R_{c}$$ (2)

Where UC_*SC*_ is the average annual cost per VMMC in SDP *s*, in country *c*. T_sc_, O_sc_, and U_sc_ correspond to facility type, ownership, and urbanicity, respectively, for SDP *s*, in country *c*. GDP_c_ is the 2016 GDP per capita in country c, Cov_c_ is the VMMC coverage (proportion of adult men covered by MC) in country c, and R_c_ is a health personnel salary index in country c taken from [25].

We validated the extrapolations at the country level by iteratively removing one country from the dataset, running the regression in Eq 2 with the remaining data, predicting the unit costs for all SDPs in the excluded country using the results from our regression, and then comparing the predicted values to the observed values. Finally, we computed several measures of performance of these comparisons to evaluate the accuracy of the extrapolations.

## Results

### Data

Table 1 shows the descriptive statistics of the primary, secondary, and pooled data sets. We present primary data both at the facility level (column 1), and the SDP level (column 2). Secondary data exists only at the SDP level (column 3). There are 220 observations in the primary dataset, which represent 38 SDP scenarios once we collapsed the data. We obtained an additional nine SDP observations from secondary data. Once we pooled both sources, there are 47 observations at the SDP level (column 4).

**Table 1:** Description of data–primary, secondary and pooled data.

	(1) Primary data	(2) Aggregated primary data^1^	(3) Secondary Data^2^	(4) Pooled data^3^
Observations	220	38	9	47
**Urbanicity**				
Rural Facilities (%)	50	50	23	45
Urban Facilities (%)	50	50	77	55
**Ownership**				
Private facilities (%)	36	36	33	36
Public facilities (%)	64	64	67	64
**Facility type**				
Hospitals (%)	47	53	66	55
Clinics (%)	53	47	34	45
**Outreach**				
Outreach (%)	13	--	--	--
Fixed (%)	87	--	--	--
Unit cost^4^ (SD^5^)	66(59)	64(48)	69(44)	65(47)
Average number of VMMC per year (SD)	1,097(1,796)	1,212(2,056)	--	--
Average VMMC coverage (SD)	49(30)	44(30)	30(27)	41(30)
Average GDP per capita (SD)	2,252(1,893)	1,995(1,813)	2,736(2,786)	2,137(2,020)
Median of data collection year	2013	2012	2013	2013
Average health personnel salary index^6^ (SD)	0.32(0.14)	0.3(0.13)	0.28(0.1)	0.3(0.13)
Number of studies	7	7	9	16
Average number of facilities per observation	1	6	5	5
Number of countries represented	8	8	6	10

Notes.

1. Aggregated primary data, collapsed at the SDP level.

2. Unit cost observations extracted from the literature review.

3. Combined aggregated primary data and secondary data (columns 2 + 3).

4. Unit costs are reported in 2016 USD.

5. Standard deviation.

6. Compensation index estimated by The International Comparison Program (ICP) using purchasing power parities (PPPs) to compare the size and price (wage) levels of health personnel around the world [25].

Collapsing primary data did not significantly change the average unit cost per VMMC (USD 66 at the facility level vs. USD 64 at the SDP level). The average unit cost from secondary data is also similar to that of the primary data (USD 69). The distribution of the sample concerning urbanicity, ownership, and type of facility was also stable (columns 1 and 2). While the sample is balanced in terms of urbanicity in primary data, the majority of the facilities in secondary data belong to urban areas (77%). Private facilities represented 37% and 33% in primary and secondary data, respectively. Finally, there is a balanced proportion of hospitals and clinics in primary data (52% and 47%, respectively), whereas hospitals are overrepresented in secondary data (66%).

The main limitation of the secondary data was that the number of VMMCs performed per year (scale) was infrequently reported in the papers. However, we were able to include unit costs from two additional countries using this source in the pooled data.

We found substantial variation in the average cost per male circumcision, both within and across countries. South Africa showed the highest within-country unit cost variation, as well as the highest median unit cost. Uganda and Namibia showed the minimum within-country unit cost variation, and the lowest median VMMC unit cost was observed in Uganda. We present the distribution of unit costs for each country in S3 Fig.

### Determinants of VMMC unit cost variation—Estimation of unit cost curves

In this section, our primary objective is to describe the association between VMMC unit costs and scale–measured by the annual number of circumcisions performed per facility. Secondly, we explored how facility-level characteristics mediate this relationship.

Table 2 presents the results of OLS (column 1) and GLM (column 2) regression models with the facility-level unit cost per circumcision as the dependent variable and the scale and facility-level features, as well as country-level per capita GDP as independent variables. The models control for the year in which each study’s data collection took place to capture possible technological progress or system-level experience. We determined the model specification with the best fit using AIC and BIC statistical criteria. The OLS model showed a R-squared parameter of 0.56.

**Table 2:** Regression models on the determinants of VMMC unit cost variations. The Dependent Variable is the Logarithm of the Facility-Level VMMC Unit Cost.

Variables	(1) OLS^1^	(2) GLM^2^
Scale (log of annual number of VMMC)	-0.13***	-0.15***
	(0.03)	(0.03)
Scale^2^ (log^2^ of annual number of VMMC)	-0.007	-0.008
	(0.01)	(0.01)
GDP per capita 2016 (log)	0.56***	0.49***
	(0.06)	(0.06)
Urbanicity (Rural = 1, Urban = 0)	0.01	0.004
	(0.07)	(0.09)
Ownership (Private = 1, Public = 0)	0.27**	0.16
	(0.12)	(0.13)
Type of Facility (Hospital = 1, Clinic = 0)	0.61***	0.62***
	(0.11)	(0.11)
Outreach (Outreach = 1, Fixed = 0)	0.27**	0.29**
	(0.12)	(0.13)
Year of data collection (Ref: 2016)	0.14***	0.18***
	(0.02)	(0.03)
Type of facility * Ownership	-0.52***	-0.53***
	(0.16)	(0.18)
Constant	4.13***	4.49***
Observations (Number of facilities)	220	220

Notes.

Standard errors in parentheses.

Significance levels

*** p<0.01

** p<0.05

* p<0.1.

1. Ordinary Least Squares regression.

2. Generalized Linear Model. The model used a gamma family and log link function.

In both models, the coefficient for scale shows a significant and negative association with unit costs, which is consistent with economies of scale; for every 10% increase in the annual number of male circumcisions, the unit costs were 1.5% lower on average. The coefficient for scale squared was not statistically significant.

The coefficient for the country-level indicator GDP per capita is statistically significant, with a positive association with unit costs. For a 10% increase in annual GDP per capita, there was an associated 5% higher unit cost, on average, holding everything else constant.

To assess the influence of urbanicity, ownership, and type of facility on unit costs, we assume that those characteristics shift the unit cost curve with respect to scale. While the coefficient of urbanicity is not statistically significant, the coefficient for ownership shows a potitive association between unit costs and private facilities compared to public ones–an average 25% higher unit costs in the former compared to the latter. The type of facility also shifts upwards the cost curve–about 60% in hospitals compared to clinics. Facilities conducting outreach are, on average, 30% costlier than those with fixed services. We also observed a positive time trend in unit costs, with a 15% average increase per year.

**Fig 1:**
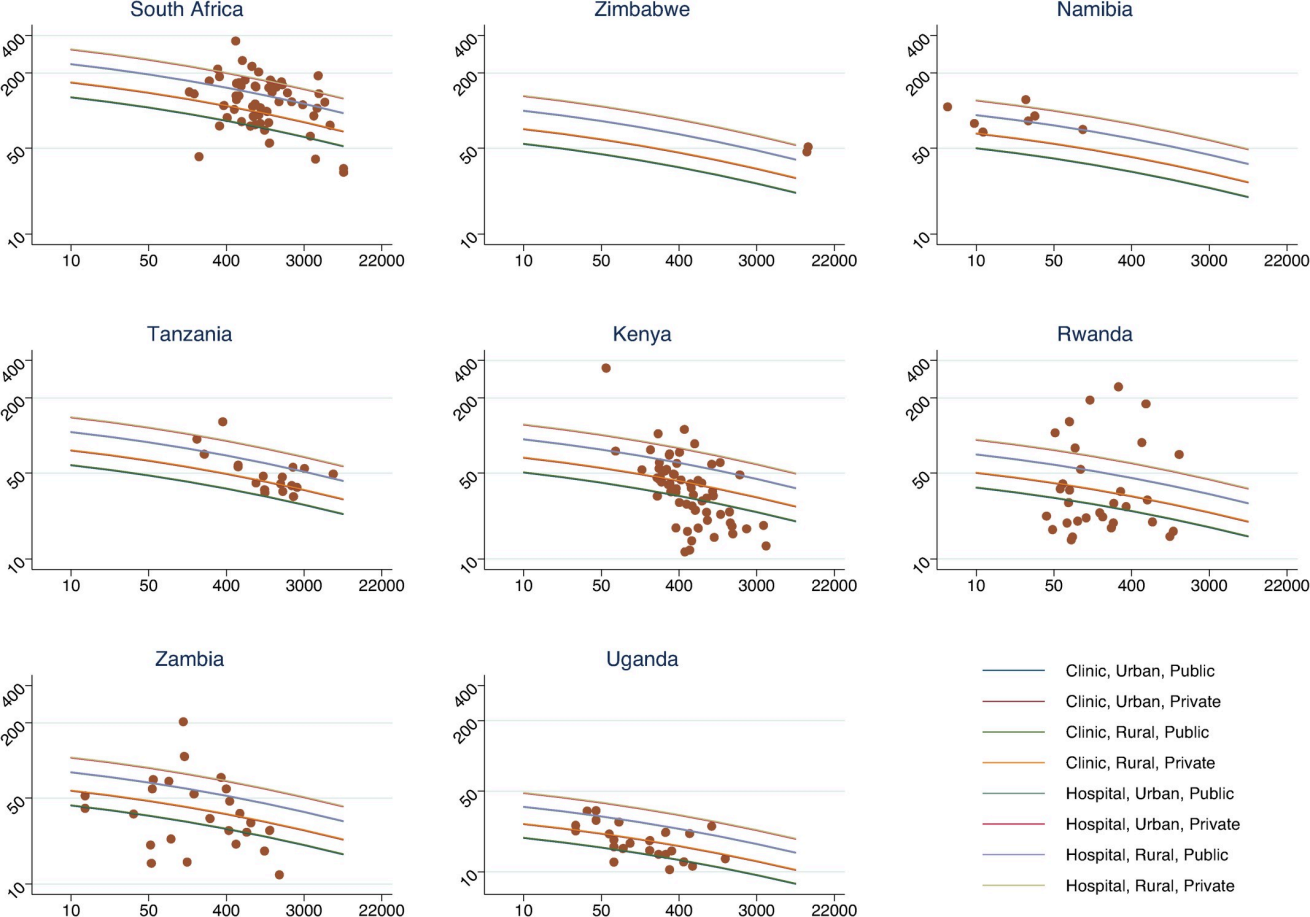
VMMC unit cost curves by country. Notes. Each panel presents the estimated cost curves for each service delivery platform defined as the combination of facility type, urbanicity, and ownership for each country, as indicated by the color guide. The panels also present the observed values of unit costs for each country.

In Fig 1, we display the results of the OLS regression model presented in Table 2, graphically. We show the results for the full sample and by country–we ordered countries by magnitude of VMMC unit costs from the most expensive (South Africa) to the least expensive (Uganda).

The unit cost curves describe the relationship between unit costs and scale resulting from the regression results. Each line represents a facility-level unit cost curve for a specific SDP. The curves shift upwards and downwards depending on the combination of implementing features. Given that the interaction between scale and GDP was not statistically significant, all curves are parallel.

The dots on the graphs represent the empirically measured facility-level unit costs–i.e., each dot represents a facility in the sample, and the observed unit cost and specific level of scale observed in the facility determine their position in the graph. The extent to which the curves (and their confidence intervals, not shown) overlap with the individual facilities, varies by country. However, most of the dots do overlap with the position of the curves, with Tanzania showing the best fit and Rwanda the worst.

This result is likely related to the fact that some countries experienced considerably more within heterogeneity, such as Zambia and Rwanda. Another critical aspect is the sample size per country. As indicated in the graphs, our primary dataset contained relatively large samples of facilities for some countries (South African, Kenya, Rwanda, Zambia, and Uganda), while relatively small samples for other (Zimbabwe, Namibia, and Tanzania).

Since our variable for urbanicity has an almost negligible influence on costs, overall, the curves for urban vs. rural scenarios overlap. However, other reported characteristics have a visibly higher impact. Private clinics show the highest costs, followed by public and private hospitals. Public clinics are the least costly facilities.

### Extrapolations of VMMC unit costs

Table 3 presents the results of the extrapolation models using the pooled dataset at the SDP-level. We tested both OLS and GLM regression models. To identify the best-performing specification of the model, we tested several alternatives and then reviewed BIC and AIC parameters to evaluate model fit. Overall, we found minimal differences between the OLS and the GLM results. OLS produced lower standard errors than GLM. Since extrapolation models are not necessarily the best-fitted models, we also tested the performance of predictions [26], and OLS predictions were, on average, more accurate than GLM’s.

**Table 3:** Extrapolation regression models–dependent variable is SDP-Level VMMC unit cost.

Variables	(1) OLS^1^	(2) GLM^2^
GDP per capita 2016 (log)	0.47***	0.44***
	(0.09)	(0.08)
Urbanicity (Rural = 1, Urban = 0)	0.10	0.10
	(0.11)	(0.13)
Ownership (Private = 1, Public = 0)	0.14	0.09
	(0.11)	(0.14)
Type of Facility (Hospital = 1, Clinic = 0)	0.14	0.10
	(0.12)	(0.13)
Health Sector Salary Index (USD)^3^	2.55***	2.84***
	(0.75)	(0.60)
National VMMC coverage	0.10	0.06
	(0.20)	(0.24)
Constant	-0.51	-0.23
	(0.51)	(0.60)
**Observations (SDP**^**4**^**)**	47	47
**R-squared**	0.68	

Notes.

Standard errors in parentheses.

Significance levels

*** p<0.01

** p<0.05

* p<0.1.

1. Ordinary Least Squares regression.

2. Generalized Linear Model. The model used a gamma family and log link function.

3. Compensation index estimated by The International Comparison Program (ICP) using purchasing power parities (PPPs) to compare the size and price levels of health personnel around the world [25].

4. Service Delivery Platform (SDP) is any given combination of three categories (facility type, urbanicity and ownership) in each country in our sample.

We found the coefficients of GDP per capita and the HSSI to be highly significant. Both are positively associated with higher costs, which supports the conventional expectations that countries with higher socioeconomic status and higher wages exhibit higher VMMC unit costs. Although the coefficients for the other independent variables were not statistically significant, we still used those coefficients for the extrapolations. Overall, the OLS model explained 69% of the variability in costs across SDP.

**Fig 2:**
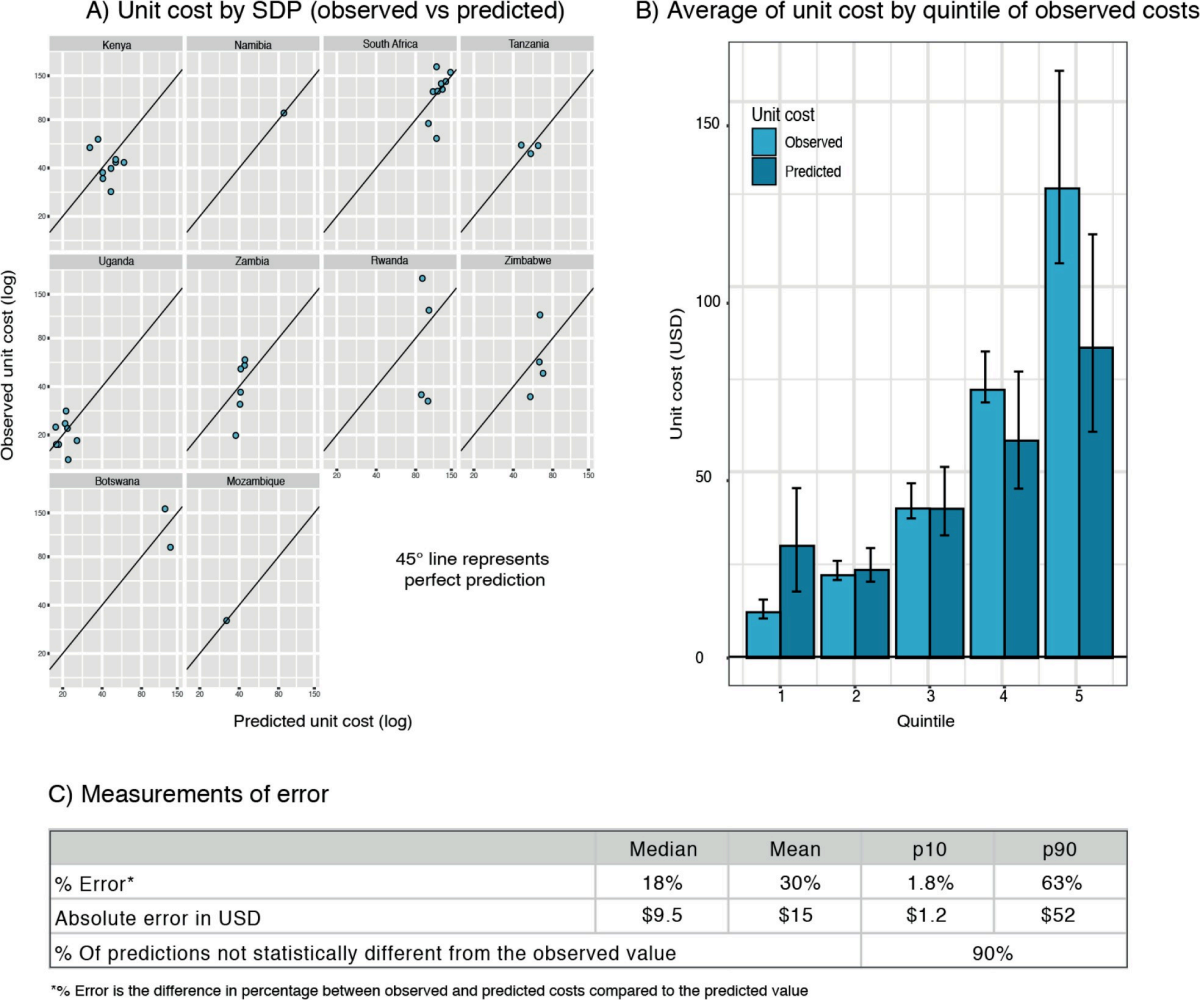
Validation of extrapolated VMMC unit cost.

To test the validity of the extrapolation models, we assessed the accuracy of the predicted unit costs. As explained before, in an iterative process, we removed all the observations of one country from the sample, then we re-ran the regression model to obtain new coefficients, and predicted the unit costs for each removed country. Finally, we compared these predictions to the observed values. We followed this process for each country in the sample. The results are presented in Fig 2. For each country, the vertical axis measures the observed unit cost, and the horizontal axis the predicted unit cost. The 45-degree line identifies perfect predictions. Overall, 90% of our predictions were not statistically different from the observed values, and the median error (in dollars) was USD 9.5, which represents 14% of the average unit cost (USD 65). Errors were higher as the unit costs increased. Using this approach, we produced estimates for all possible SDPs (112 combinations) in the 14 priority countries for VMMC in Africa, where only 10% of the SDPs had published estimations. We present the estimates in S2 Table.

## Discussion

This analysis builds upon published VMMC studies and available facility level data to create robust cost estimates for VMMC services in Sub-Saharan Africa. Using data from 16 VMMC studies, we developed econometric models to estimate unit cost curves for VMMC services in a variety of clinical and geographical settings. By identifying key cost drivers, including country- and facility-level characteristics, as well as the effects of scale, we projected VMMC unit costs for several implementation settings, including many for which there are currently no such data. Furtermore, our approach produces unit costs estimates with variance parameters, which allows to explicitly account for uncertainty, unlike the currently used approach which overwhelmingly relies on point estimates with no information on uncertainty.

We studied unit costs in two distinct ways. First, we modeled VMMC unit costs in a sample of 220 facilities in eight countries as a function of a variety of facility-level characteristics or service delivery models (SDM), namely the type of facility, urbanicity, ownership, and scale (number of VMMC performed per year). Secondly, we extrapolated unit cost estimates for countries outside our sample and validated our predictions systematically in ten countries. Overall, our results showed a high level of accuracy, with a median percentage error of 18% between observed and predicted costs.

While our study did not produce new empirical data, our results did increase by a tenfold the availability of unit costs estimates for 128 SDPs in 14 priority countries for VMMC. It is to our knowledge, the most comprehensive analysis of VMMC unit costs to date. Furthermore, we provide a proof of concept of the ability to generate predictive cost estimates for settings where empirical data does not exist, leveraging previously published studies. Our relatively large and diverse sample size enabled us to develop statistically significant cost estimates accounting for a variety of context characteristics while taking into account the effect of scale on cost variations. The latter is a crucial economic aspect largely absent in most of the costing literature. Our approach contributes not only with additional information for VMMC programs but also serves as a guide for using existing cost data to create econometric models of unit costs for other interventions and diseases.

Cost-effective interventions are essential in maximizing the impact of limited resources in curbing the HIV epidemic in sub-Saharan Africa. Furthermore, funders, policy-makers, and decision-makers all need accurate data on the economics of program implementation to inform decisions. A timely, reliable, and precise description of the unit costs of services is, therefore, a critical resource for them. Our research also has the potential to inform researchers developing mathematical models on the impact of the HIV response on curbing the epidemic and reaching global targets of coverage and impact.

One goal was to identify which facility-level variables significantly influence VMMC unit costs. We were limited in this respect by the subset of variables included in all studies. Despite this limitation, we were able to analyze critical facility-level characteristics. Overall, the most statistically significant features our analysis found were the type of facility (hospital vs. clinic), ownership (public vs. private), scale, and the year of data collection. Urbanicity and whether or not the facility conducted outreach showed no statistically significant association with unit cost variations.

Consistent with previous research [27–29], we found evidence consistent with the existence of economies of scale in the provision of VMMC services–decreasing unit costs as the number of services produced increased. The correlation was robust and statistically significant across all the models tested, with an estimated 12% to 22% reduction in unit costs associated with a ten percent increase in the size of service production. The lack of significance in the coefficient of the squared term of the scale indicates that the percentage of reduction in unit cost remains constant throughout the range of our data. Understanding the interaction between unit costs and scale and moving away from the misleading notion of "the unit cost" as a fixed feature of health services is vital.

### Limitations

Readers should interpret the results from this study in light of several limitations. The primary shortcoming stems from the fact that we used exclusively previously published data, and we could not control study design, measurement methods, or the analysis and estimation of unit costs. The implications are potentially significant. The most immediate implication is that we inherited the flaws and limitations of the original studies. The design of the research projects included in the analysis necessarily constrained our results’ external and internal validity. Secondly, the studies’ quality is most likely uneven, resulting in systematic bias across countries. Third, relying on previously published studies also implies that we combined observations not originally meant to be analyzed together, which has statistical implications and means that measurement methods were not necessarily consistent. Different measurement and estimation methods could compromise comparability across studies. Fourth, the final sample is constrained to the countries and programs studied and published before. We addressed these challenges in various steps. We addressed the first, second, and third implications mentioned above by using a systematic literature review to select studies. We used inclusion and exclusion criteria to ensure a minimum level of quality in the studies selected and criteria to warrant comparability of the results, such as rigorous measurement methods and exclusion of non-empirical cost estimations. We discarded several studies and observations from the analysis due to ambiguous, unclear, or inconsistent definitions reported by original study authors. We also followed a rigorous process to standardize the definitions and measurement of all variables included in the analyses, including categories of inputs and costs, total costs, and facility characteristics. The fourth implication above is impossible to address directly; however, the extrapolation analysis’s objective was precisely to fill such gaps in data.

Another limitation of the study is that our definition of "service delivery platforms" was constrained by the characteristics measured by all the original studies; therefore, we could have omitted characteristics relevant for efficiency not captured in our data. We used combinations of type of facility, urbanicity, and ownership to define platforms. To the extent that other features are critical for efficiency and, therefore, for unit costs, our definition is incomplete and our results potentially biased. For example, we did not include staff size and staff composition measures, which are associated with unit costs heterogeneity [4]. Nevertheless, such characteristics are probably also correlated with the ones we did include in the analysis, attenuating this omission. Furthermore, the extrapolation validation suggests that our approach is robust since the models using our SDP definition successfully predict unit costs in countries excluded from the sample.

Another limitation was the unbalanced sample size per country in our datasets (both primary and secondary). For some countries, like Kenya and South Africa, our data included large samples, while for other countries, such as Zimbabwe and Namibia, we had access to only a small number of facilities. The implication of this limitation is statistical, yielding different levels of uncertainty in our estimations across settings. Once more, the validation of VMMC unit costs’ extrapolations suggests this limitation did not compromise our approach’s predictive ability.

Finally, our extrapolation models were limited to only a few characteristics describing both the context and the country’s health system. Although the results show that our approach performed well, more sophisticated approaches might produce better extrapolations in future works. We used country-level characteristics describing the economy (GDP per capita and health sector-specific salary indexes), the health system (VMMC coverage), and the epidemic (HIV prevalence) to extrapolate unit costs to countries not included in our sample. We tested several model specifications and chose the most robust one. However, the model specifications we used are potentially simplistic and are undoubtedly subject to improvement as richer data sources become available. We hope that future exercises can expand and improve upon our approach.

In spite of these limitations, we were able to successfully standardized disparate data sets and produce robust cost estimates for VMMC programs across the 13 critical VMMC countries in sub-Saharan Africa. This study provides reliable cost estimations for a wide variety of settings and can be used to help guide ongoing scale-up efforts of VMMC services in Africa. Additionally, by applying the predictive cost modeling approach explored in this paper, we open up the potential to expand our research to other interventions, providing accurate, timely cost data to the program planners, policy-makers, and funders who need it.

### Policy implications

Cost-effective interventions are essential to maximizing the impact of limited resources on curbing the HIV epidemic in sub-Saharan Africa. Furthermore, funders, policy-makers, and decision-makers all need accurate data on program implementation costs and efficiency to inform decisions. Therefore, a timely, reliable, and accurate description of services’ unit costs is a critical resource for them.

Our project contributes to filling the gaps in cost data in two ways. First, filling blanks; of the 14 priority countries, there was no available data at the moment of our literature review for 8 of them (S2 Table). This paper’s supplemental material provides unit costs for all 14 priority countries. Program managers there can use this information for planning and monitoring purposes. Secondly, our approach produces a rich data set of unit costs for all the countries in the priority list and three service delivery modalities within each country. Using this rich source of unit costs, program directors can access more specific and nuanced cost information than previously. The cost of WMMC is not only not fixed, but it is determined by critical aspects of service delivery that our study explicitly accounts for and examines.

Finally, our research can also be helpful for researchers developing mathematical models of the HIV response. In cost-effectiveness analyses, estimations usually rely on highly sophisticated epidemiological modeling to predict the effectiveness side of the equation. Simultaneously, making overly simplistic assumptions on costs -, many times a single number for each scenario or policy alternative. This approach’s result is most likely flawed because it depends on both the cost and the effectiveness estimations. Our results provide a rich dataset of unit cost estimations (and confidence intervals) for three SDM in 14 countries and a model specification to reproduce the results. We hope that this exercise will help improve modeling efforts in the future.

## Supporting information

S1 Fig Flow chart for obtaining the analytical sample. (PDF)

S2 Fig Process of collapsing facility-level primary data unit costs. (DOCX)

S3 Fig Distribution of facility-level unit costs per VMMC, by country. (DOCX)

S1 Table List of combinations of service delivery platforms (SDP). (DOCX)

S2 Table Estimations for all possible SDPs (112 combinations) in the 14 priority countries for VMMC in Africa. (XLSX)

S1 File Data obtainment process and standardization. (DOCX)

## Abbreviations

GHCC: Global Health Cost Consortium
GLM: Generalized Linear Models
OLS: Ordinary Least Squares
SDP: Service Delivery Platform
VMMC: Voluntary Medical Male Circumcision

## References

[1] WHO. HIV/AIDS factsheet. Geneva, Switzerland; 2019.

[2] UNAIDS. UNAIDS Data 2017. Joint United Nations Programme on HIV/AIDS (UNAIDS). 2017. 12349391

[3] Bautista-Arredondo S, Colchero MA, Amanze OO, La Hera-Fuentes G, Silverman-Retana O, Contreras-Loya D, et al. Explaining the heterogeneity in average costs per HIV/AIDS patient in Nigeria: The role of supply-side and service delivery characteristics. PLoS One. 2018;13: e0194305. 10.1371/journal.pone.0194305 29718906 PMC5931468

[4] Bautista-Arredondo S, Sosa-Rubi SG, Opuni M, Contreras-Loya D, La Hera-Fuentes G, Kwan A, et al. Influence of supply-side factors on voluntary medical male circumcision costs in Kenya, Rwanda, South Africa, and Zambia. PLoS One. 2018;13: e0203121–e0203121. 10.1371/journal.pone.0203121 30212497 PMC6136711

[5] Bautista-Arredondo S, Hera-Fuentes G La, Contreras-Loya D, Kwan A, Janae Van Buren S, Amanze OO, et al. Efficiency of HIV services in Nigeria: Determinants of unit cost variation of HIV counseling and testing and prevention of mother-to-child transmission interventions. PLoS One. 2018. 10.1371/journal.pone.0201706 30192765 PMC6128456

[6] Pineda-Antunez C, Martinez-Silva G, Cerecero-Garcia D, Alexander L, Cameron DB, Chiwevu C, et al. Meta-analysis of average costs of HIV testing and counselling and voluntary medical male circumcision across thirteen countries. African J AIDS Res. 2019. 10.2989/16085906.2019.1679850 31779565

[7] Cerecero-García D, Pineda-Antunez C, Alexander L, Cameron D, Martinez-Silva G, Obure CD, et al. A meta-analysis approach for estimating average unit costs for ART using pooled facility-level primary data from African countries. African J AIDS Res. 2019. 10.2989/16085906.2019.1688362 31779577

[8] UNAIDS. Manual for costing HIV facilities and services. Geneva, Switzerland; 2011.

[9] DeCormier Plosky W, Bollinger LA, Alexander L, Cameron DB, Carroll LN, Cunnama L, et al. Developing the Global Health Cost Consortium Unit Cost Study Repository for HIV and TB: methodology and lessons learned. African J AIDS Res. 2019;18: 263–276. 10.2989/16085906.2019.1680398 31779571

[10] Vassall A, Sweeney S, Kahn J, Gomez GB, Bollinger L, Marseille E, et al. Reference Case for Estimating the Costs of Global Health Services and Interventions. 2017.

[11] Auvert B, Taljaard D, Lagarde E, Sobngwi-Tambekou J, Sitta R, Puren A. Randomized, controlled intervention trial of male circumcision for reduction of HIV infection risk: The ANRS 1265 trial. PLoS Med. 2005;2: 1112–1122. 10.1371/journal.pmed.0020298 16231970 PMC1262556

[12] Binagwaho A, Pegurri E, Muita J, Bertozzi S. Male Circumcision at Different Ages in Rwanda: A Cost-Effectiveness Study. Kalichman SC, editor. PLoS Med. 2010;7: e1000211. 10.1371/journal.pmed.1000211 20098721 PMC2808207

[13] Bailey RC, Moses S, Parker CB, Agot K, Maclean I, Krieger JN, et al. Male circumcision for HIV prevention in young men in Kisumu, Kenya: a randomised controlled trial. Lancet. 2007;369: 643–656. 10.1016/S0140-6736(07)60312-2 17321310

[14] Tobian AAR, Kacker S, Quinn TC. Male Circumcision: A Globally Relevant but Under-Utilized Method for the Prevention of HIV and Other Sexually Transmitted Infections. Annu Rev Med. 2014;65: 293–306. 10.1146/annurev-med-092412-090539 24111891 PMC4539243

[15] Wawer MJ, Tobian AA, Kigozi G, Kong X, Gravitt PE, Serwadda D, et al. Effect of circumcision of HIV-negative men on transmission of human papillomavirus to HIV-negative women: A randomised trial in Rakai, Uganda. Lancet. 2011;377: 209–218. 10.1016/S0140-6736(10)61967-8 21216000 PMC3119044

[16] Uthman OA, Popoola TA, Uthman MMB, Aremu O. Economic evaluations of adult male circumcision for prevention of heterosexual acquisition of HIV in men in sub-Saharan Africa: A systematic review. PLoS One. 2010;5. 10.1371/journal.pone.0009628 20224784 PMC2835757

[17] UNAIDS. Joint Strategic Action Framework to Accelerate the Scale-Up of Voluntary Medical Male Circumcision for HIV Prevention in Eastern and Southern Africa, 2012–2016. Geneva; 2011.

[18] Davis SM, Hines JZ, Habel M, Grund JM, Ridzon R, Baack B, et al. Progress in voluntary medical male circumcision for HIV prevention supported by the US President’s Emergency Plan for AIDS Relief through 2017: Longitudinal and recent cross-sectional programme data. BMJ Open. BMJ Publishing Group; 2018. p. e021835. 10.1136/bmjopen-2018-021835 PMC6120649 30173159

[19] Schwartländer B, Stover J, Hallett T, Atun R, Avila C, Gouws E, et al. Towards an improved investment approach for an effective response to HIV/AIDS. Lancet. 2011;377: 2031–2041. 10.1016/S0140-6736(11)60702-2 21641026

[20] Holmes KK, Bertozzi S, Bloom BR, Jha P, Gelband H, DeMaria LM, et al. Major Infectious Diseases: Key Messages from Disease Control Priorities, Third Edition. Disease Control Priorities, Third Edition (Volume 6): Major Infectious Diseases. The World Bank; 2017. pp. 1–27. 10.1596/978-1-4648-0524-0_ch1 30212102

[21] The World Bank. GDP deflator (base year varies by country). 2017. Available: https://data.worldbank.org/indicator/NY.GDP.DEFL.ZS.

[22] Cameron DB, Mustafa Diab M, Carroll LN, Bollinger LA, DeCormier Plosky W, Levin C, et al. The state of costing research for HIV interventions in sub-Saharan Africa. African J AIDS Res. 2019;18: 277–288. 10.2989/16085906.2019.1679200 31779568

[23] Menzies NA, Berruti AA, Blandford JM, Cohen M, Chen Y, McCauley M, et al. The Determinants of HIV Treatment Costs in Resource Limited Settings. Fox MP, editor. PLoS One. 2012;7: e48726. 10.1371/journal.pone.0048726 23144946 PMC3492412

[24] Guthery FS, Burnham KP, Anderson DR. Model Selection and Multimodel Inference: A Practical Information-Theoretic Approach. J Wildl Manage. 2003;67: 655. 10.2307/3802723

[25] World Bank. Purchasing Power Parities and the Real Size of World Economies: A Comprehensive Report of the 2011 International Comparison Program. Washington, DC: World Bank. The World Bank; 2014. 10.1596/978-1-4648-0329-1

[26] Shmueli G. To Explain or To Predict? Stat Sci. 2010;25: 289–310. 10.2139/ssrn.1351252

[27] Bautista-Arredondo S, Sosa-Rubi SG, Opuni M, Contreras-Loya D, Hera-Fuentes G La, Kwan A, et al. Influence of supply-side factors on voluntary medical male circumcision costs in Kenya, Rwanda, South Africa, and Zambia. PLoS One. 2018. 10.1371/journal.pone.0203121 30212497 PMC6136711

[28] Tchuenche M, Njeuhmeli E, Schütte C, Ngubeni L, Choge I, Martin E, et al. Voluntary medical male circumcision service delivery in South Africa: The economic costs and potential opportunity for private sector involvement. PLoS One. 2018;13: 1–15. 10.1371/journal.pone.0208698 30557330 PMC6296535

[29] Menon V, Gold E, Godbole R, Castor D, Mahler H, Forsythe S, et al. Costs and impacts of scaling up voluntary medical male circumcision in Tanzania. PLoS One. 2014;9. 10.1371/journal.pone.0083925 24802022 PMC4011575

