# Supplementary material for: Moving away from the "unit cost". Predicting country-specific average cost curves of VMMC services accounting for variations in service delivery platforms in sub-Saharan Africa
Source: PLoS One. 2021 Apr 22;16(4):e0249076. doi: 10.1371/journal.pone.0249076 (PMC8062035; doi:10.1371/journal.pone.0249076)
Supplement: S2 Fig — (DOCX) [file pone.0249076.s002.docx]

**S3 Annex.** Process of collapsing facility-level primary data unit costs.

Collapsing is done by averaging unit costs for facilities with the same set of characteristics.

Note: these numbers are examples only and do not reflect the actual data in our data sets.
