# Supplementary material for: Moving away from the "unit cost". Predicting country-specific average cost curves of VMMC services accounting for variations in service delivery platforms in sub-Saharan Africa
Source: PLoS One. 2021 Apr 22;16(4):e0249076. doi: 10.1371/journal.pone.0249076 (PMC8062035; doi:10.1371/journal.pone.0249076)
Supplement: S3 Fig — (DOCX) [file pone.0249076.s003.docx]

S5 Annex. Distribution of facility-level unit costs per VMMC, by country
