# Supplementary material for: Moving away from the "unit cost". Predicting country-specific average cost curves of VMMC services accounting for variations in service delivery platforms in sub-Saharan Africa
Source: PLoS One. 2021 Apr 22;16(4):e0249076. doi: 10.1371/journal.pone.0249076 (PMC8062035; doi:10.1371/journal.pone.0249076)
Supplement: S1 Table — (DOCX) [file pone.0249076.s004.docx]

## S4 Annex. List of combinations of Service Delivery Platforms (SDP)

| **Country** | **Urbanicity** | **Ownership** | **Facility Type** |
| --- | --- | --- | --- |
| Kenya | Urban | Public | Clinic |
| Kenya | Urban | Public | Hospital |
| Kenya | Urban | Private | Clinic |
| Kenya | Urban | Private | Hospital |
| Kenya | Rural | Public | Clinic |
| Kenya | Rural | Public | Hospital |
| Kenya | Rural | Private | Clinic |
| Kenya | Rural | Private | Hospital |
| Namibia | Urban | Public | Hospital |
| South Africa | Urban | Public | Clinic |
| South Africa | Urban | Public | Hospital |
| South Africa | Urban | Private | Clinic |
| South Africa | Urban | Private | Hospital |
| South Africa | Rural | Public | Clinic |
| South Africa | Rural | Public | Hospital |
| South Africa | Rural | Private | Clinic |
| South Africa | Rural | Private | Hospital |
| Tanzania | Urban | Public | Clinic |
| Tanzania | Urban | Public | Hospital |
| Tanzania | Rural | Public | Hospital |
| Uganda | Urban | Public | Hospital |
| Uganda | Urban | Private | Clinic |
| Uganda | Urban | Private | Hospital |
| Uganda | Rural | Public | Clinic |
| Uganda | Rural | Public | Hospital |
| Uganda | Rural | Private | Clinic |
| Uganda | Rural | Private | Hospital |
| Zambia | Urban | Public | Clinic |
| Zambia | Urban | Public | Hospital |
| Zambia | Urban | Private | Clinic |
| Zambia | Rural | Public | Clinic |
| Zambia | Rural | Public | Hospital |
| Zambia | Rural | Private | Clinic |
| Rwanda | Urban | Public | Clinic |
| Rwanda | Urban | Public | Hospital |
| Rwanda | Rural | Public | Clinic |
| Rwanda | Rural | Public | Hospital |
| Zimbabwe | Rural | Public | Hospital |
| Botswana | Rural | Public | Clinic |
| Botswana | Urban | Public | Hospital |
| Kenya | Urban | Private | Hospital |
| Kenya | Urban | Private | Hospital |
| Mozambique | Urban | Public | Hospital |
| Mozambique | Urban | Public | Hospital |
| South Africa | Urban | Public | Hospital |
| South Africa | Urban | Public | Hospital |
| Uganda | Urban | Private | Hospital |
| Uganda | Urban | Private | Hospital |
| Zimbabwe | Rural | Private | Clinic |
| Zimbabwe | Urban | Public | Hospital |
| Zimbabwe | Urban | Public | Hospital |
| Zimbabwe | Urban | Public | Hospital |
| Zimbabwe | Urban | Public | Clinic |
| Zimbabwe | Urban | Public | Hospital |
| Zimbabwe | Urban | Public | Hospital |
