# Supplementary material for: Moving away from the "unit cost". Predicting country-specific average cost curves of VMMC services accounting for variations in service delivery platforms in sub-Saharan Africa
Source: PLoS One. 2021 Apr 22;16(4):e0249076. doi: 10.1371/journal.pone.0249076 (PMC8062035; doi:10.1371/journal.pone.0249076)
Supplement: S1 File — (DOCX) [file pone.0249076.s006.docx]

**S2 Annex. Data obtainment process and standardization**

The objective of this appendix is to provide an overview of the procedures used to harmonize and standardize the study-specific datasets in order to create a merged facility level primary data dataset. There are four major steps in to achieve this aim:

1. Converting each dataset into Stata 15 format
2. Filling in a study-specific codebook with key variables
3. Running a standardization code
4. Concatenating the separate cleaned datasets into one dataset

Creating a codebook of variables for datasets harmonization

**Table S1. Broad and narrow input cost categories**

| **Broad input cost categories** | **Narrow input cost categories** | **Description of inputs in input cost categories** |
| --- | --- | --- |
| Personnel | Service delivery personnel | Doctors, nurses, counsellors; Pharmacists; Lab/diagnostic personnel; Outreach workers, peer supporters, social workers; Community volunteers, or home visitors |
|  | Support personnel | Administrators, supervisors; Procurement officers, supply clerks, accountants; Legal staff; Receptionists; Social media coordinators, community strategy/mobilization supervisors; Data and IT staff; Drivers; Gardeners; Security guards; Kitchen staff; Custodians or cleaning staff. |
| Capital | Lab/ diagnostic equipment | Centrifuges, incubators, microscopes, water baths. |
|  | Equipment (medical/intervention, excl. lab) | Refrigerators, freezers, monitoring equipment; Tents. |
|  | Equipment (non-medical/intervention) | Furniture: beds, benches/couches, chairs, desks, tables, lamps/fixtures, filing/drug cabinets, bookcases; Computers, monitors, LCD projectors, printers; Software; Power outlets, or paper shredders. |
|  | Vehicles, capital | Bicycles; Motorcycles; Cars, vans or SUVs; Trucks; Boats; or Airplanes |
|  | Building/space, capital | Construction/purchased floor space in a health facility or training school; Truck containers; Storage facilities; Administrative offices; Wells; or Latrines. |
|  | Other capital | Start-up training and materials; Licenses/copyrights |
| Recurrent | Supplies (key drugs) | TB drugs; PrEP; ARVs; PEP; Hepatitis/STI/OI education; Antibiotics; or Contraceptives. |
|  | Supplies (medical/intervention, excl. key drugs) | Vaccines; Syringes, test kits, sputum bottles, speculum, cotton swabs, microscope slides reagents; Gloves, gowns, masks, bandages; Small medical equipment; or Small containers to hold drugs. |
|  | Supplies (non-medical/non-intervention) | Pens, pencils, dry-erase markers, highlighters; Printer paper, post-it notes, notebooks, calendars; Paper clips, binder clips; File folders; Envelopes, stamps; Tape, glue; Scissors, staplers, hole-punchers, calculators; Memory sticks; Batteries; or Lanyards. |
|  | Building/space | Rent for capital inputs; Maintenance: Painting, roof, heating/plumbing, windows; Tires, spare parts, oil/lubricants, tune-ups; or Computer repair. Lighting, heating, water; Telephone, or internet. |
|  | Other recurrent | Gasoline, fuel; Tolls; or Contracted transportation services; Food (at facilities/meetings; for nutritional support to improve health or lessen side effects of drugs); Vitamins, or Contracted meal services. Recurrent training; Medical malpractice insurance; Insurance for capital building, vehicles, or equipment; Registration fees for capital items, for memberships in professional organizations, or for use of copyrighted materials for communication purposes (icons, photos, etc.); Contracted services such as laboratory, storage, waste removal (even if just burning and/or burying), security, or information technology if outsourced; Courier/UPS service; or Other recurrent costs. |

Homogenizing variables from different databases

Since not all datasets will be alike, members of the GHCC Data and Analytics team worked to standardize and homogenize each dataset prior to merging them into the primary dataset. Each dataset acquired was unique in terms of variable names, variables measured and specific methods to measure each variable. We ensured that the basic elements to estimate unit costs were included in all datasets. Therefore, at the very least, datasets can be merged along a minimum set of common variables. Below are specific components to be considered in the standardization and cleaning process.

1. Standardization of datasets into a single statistical analysis software: all the data received was in excel format. Since the analyses in this paper were performed in stata software, we standardized all datasets into this format for data cleaning.
2. Identifying levels of disaggregation for each dataset: Datasets can be disaggregated in several levels (**Figure S1**):
   - Database attributes – all the cost categories included in the study (i.e. capital costs, personnel direct costs, personnel support costs, among others); the intervention or interventions costed; economic perspective, and study design (i.e. cross sectional, longitudinal)
   - Broad cost categories – Overall cost categories included in the dataset (i.e. capital costs, recurrent costs, personnel costs).
   - Narrow costs categories included within each broad cost category (e.g. Building/space, medical supplies, key drugs, vehicles)
3. Homogenizing variables: based on the defined narrow cost categories, we created a dataset with homogeneous variable names. We identified variables that are essentially the same across datasets – regardless of the variable name, using the third level of disaggregation. This identification process involved reviewing the dictionary/variable codebook that each author sent along with their datasets and comparing variable definitions among the codebooks. If variable names are distinct between datasets but the definitions are comparable, we created a dataset with homogenous variable names by relabeling the variable name in the codebook shown in Table S1. In **Figure S2** we showed the process of data standardization and merging.

**Figure S1. Levels of disaggregation within each dataset**


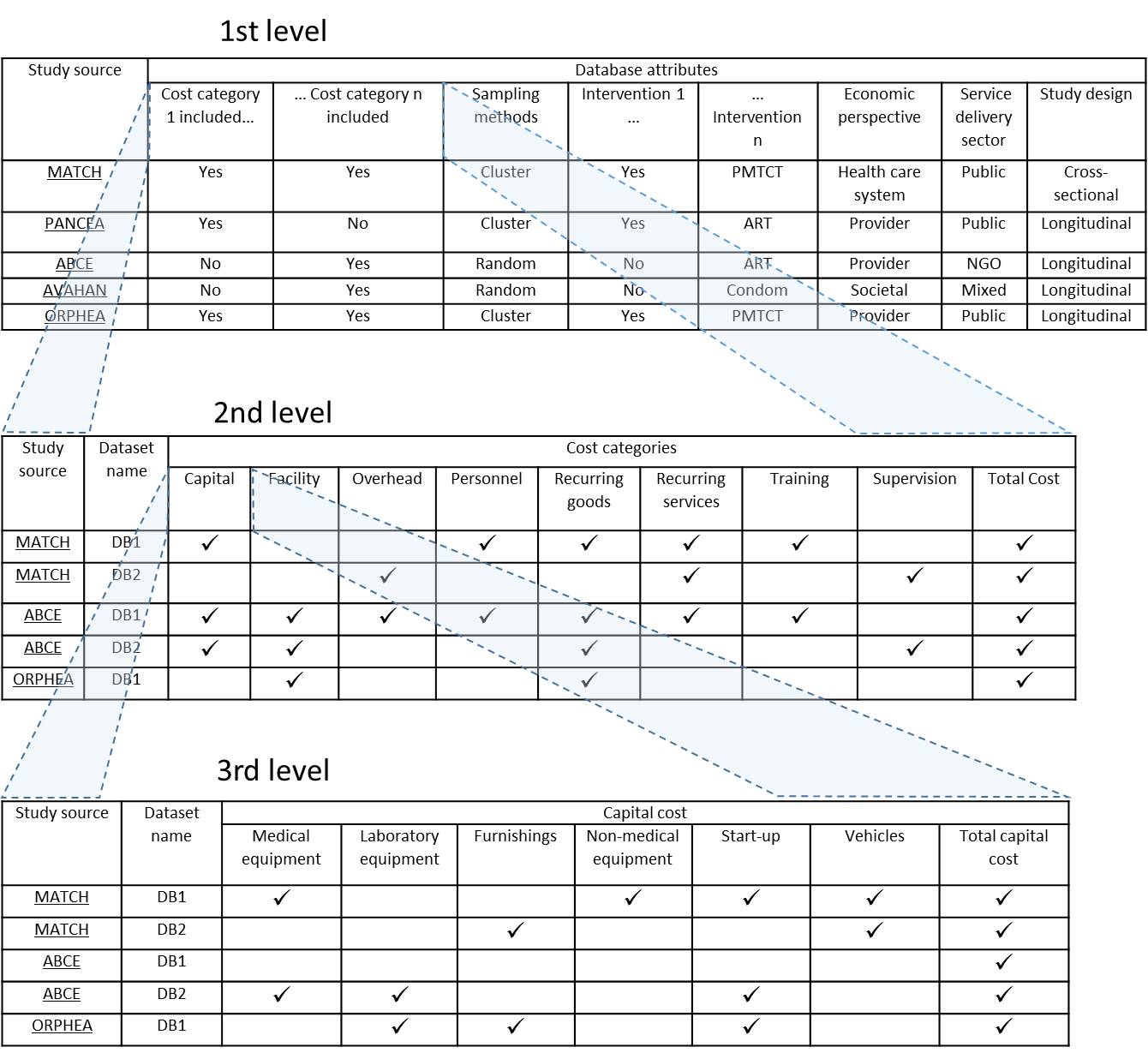


**Figure S2. Data standardization and merging**

Standardization of unit cost

To estimate total annual costs and unit costs of VMMC, we added the three broad input cost categories for each facility – capital, recurrent, and personnel. To make sure all these broad categories were comparable between studies, only common cost categories within were included (Figure S3) – we excluded cost categories included in only one study.

**Figure S3. Cost categories standardization**

Once all the studies were comparable in terms of cost categories, the total annual cost of VMMC was estimated as follows:

$$Total annual cost of VMMC=total Personnel cost+total Capital cost+total Recurrent cost$$

Then VMMC unit cost, was estimated using the total annual cost of male circumcision intervention divided by the total number of male circumcisions performed in the same facility, in the same year

$$Unit cost of VMMC=\frac{Total annual cost of VMMC}{Annual number of MC performed}$$
